# The Footprint of Microbiome in Pediatric Asthma—A Complex Puzzle for a Balanced Development

**DOI:** 10.3390/nu15143278

**Published:** 2023-07-24

**Authors:** Ancuta Lupu, Elena Jechel, Cristina Maria Mihai, Elena Cristina Mitrofan, Silvia Fotea, Iuliana Magdalena Starcea, Ileana Ioniuc, Adriana Mocanu, Dragos Catalin Ghica, Alina Popp, Dragos Munteanu, Maria Oana Sasaran, Delia Lidia Salaru, Vasile Valeriu Lupu

**Affiliations:** 1 Faculty of General Medicine, “Grigore T. Popa” University of Medicine and Pharmacy, 700115 Iasi, Romania; 2 Faculty of General Medicine, Ovidius University, 900470 Constanta, Romania; 3 CF Clinical Hospital, 700506 Iasi, Romania; 4 Clinical Medical Department, Faculty of Medicine and Pharmacy, “Dunarea de Jos” University of Galati, 800008 Galati, Romania; 5 Faculty of General Medicine, “Carol Davila” University of Medicine and Pharmacy, 020021 Bucharest, Romania; 6 Faculty of General Medicine, “George Emil Palade” University of Medicine, Pharmacy, Science and Technology, 540142 Targu Mures, Romania

**Keywords:** asthma, gut microbiota, children, dysbiosis

## Abstract

Considered to be of greater complexity than the human genome itself, the microbiome, the structure of the body made up of trillions of bacteria, viruses, and fungi, has proven to play a crucial role in the context of the development of pathological processes in the body, starting from various infections, autoimmune diseases, atopies, and culminating in its involvement in the development of some forms of cancer, a diagnosis that is considered the most disabling for the patient from a psychological point of view. Therefore, being a cornerstone in the understanding and optimal treatment of a multitude of ailments, the body’s microbiome has become an intensively studied subject in the scientific literature of the last decade. This review aims to bring the microbiome–asthma correlation up to date by classifying asthmatic patterns, emphasizing the development patterns of the microbiome starting from the perinatal period and the impact of pulmonary dysbiosis on asthmatic symptoms in children. Likewise, the effects of intestinal dysbiosis reflected at the level of homeostasis of the internal environment through the intestine–lung/vital organs axis, the circumstances in which it occurs, but also the main methods of studying bacterial variability used for diagnostic purposes and in research should not be omitted. In conclusion, we draw current and future therapeutic lines worthy of consideration both in obtaining and maintaining remission, as well as in delaying the development of primary acute episodes and preventing future relapses.

## 1. Introduction

Bronchial asthma is a chronic condition, with an increased level of incidence among pediatric patients, partly due to the physiopathological cascade that occurs as a result of the body’s adaptation to the environment but also of the variety of allergens with which they interact. The diagnostic and therapeutic lines raise the capacity of the clinician, both by the need to exclude differential diagnoses and by the desire for defensive management, with as few hospitalizations and symptomatology exacerbations as possible. An optimal therapeutic threshold is also desired to provide the best quality of life but also a satisfactory integration in the community, a particularly important aspect among children from the perspective of harmonious psycho-social development and a high therapeutic adherence [1,2].

Current research regarding the body’s homeostasis focuses on studying the interaction between the environment (exogenous or endogenous) and the host, highlighting the involvement of the microbiome in human development. It simultaneously plays the role of “gatekeeper”, forming the first physiological line of defense in terms of absorption through inhalation, ingestion, or dermal contact, thus modulating the nature and degree in which exogenous agents are absorbed and distributed in the internal environment, as well as of “watchman”, generating signals that are then directed to the organs and organ systems that are components of the physiological barriers (skin, intestine, lungs) [3,4].

The specific structures of the microbiome known to influence the appearance, development, and exacerbation of asthma at the population level, including in pediatric age groups, are represented by the microbiome of the upper respiratory tract, the gastrointestinal system, but also the microbiome of the external environment. The colonization of the upper respiratory tract seems to start very early, an aspect objectivized with the help of tracheal aspirates obtained from newborns just a few hours after birth, preparations on which are predominantly *Firmicutes* and *Proteobacteria*, in addition to *Actinobacteria* and *Bacteroidetes*, were identified [5]. Being part of a structure spread over an area of approximately 6.5 m that makes up the human digestive system, it is estimated that, at the level of the small intestine of each person, live between 150 and 400 species with variable properties from one individual to another and even in the same individual at different times of existence, the most frequent being *Bacteroidetes*, *Firmicutes*, *Actinobacteria* and *Proteobacteria phyla* [6].

## 2. Materials and Method

Considering the multitude of implications of the human microbiome in the body’s homeostasis, the response level of bronchial asthma, but also the particularities of its management in the pediatric age, we consider it appropriate to make a correlation between the two entities. For this purpose, we carried out a review of the literature from the last decades. 

Scientific articles were selected by accessing relevant databases for medical research, such as PubMed, analyzing the most recent studies in the area of interest, which we discovered by searching for phrases: “microbiome”, “pathogenesis of asthma”, “IgE”, “predisposing factors”, “pollution”, “atopies”, “childhood asthma”, “asthmatic phenotypes”, dysbiosis”, “microbial sites”, probiotic”, “prebiotic”, “symbiotic”, or “asthma therapy”. 

The included population was represented by newborns, children, and adolescents under the age of 18, in which the evolutionary description of the microbiota and the impact of dysbiosis on organic diseases, mainly asthma, were followed. The exclusion criteria were represented by study groups over 18 years old, research on small groups of patients (which do not show statistical relevance), and studies that lost a large number of subjects or that may be associated with risks of bias. For a complete presentation, the inclusion of data on maternal diet, peri- and post-natal factors, and the effect of probiotic, prebiotic, or symbiotic supplementation among infants and mothers during pregnancy and breastfeeding was considered. In addition, where the current literature is not fully developed regarding children, future research directions have been drawn by referring to the results obtained on groups of adults.

The extracted data concerns the type of study performed, the size of the patient group, the geographical location, and the conclusions of the study, but also theoretical and practical aspects derived from it.

## 3. Epidemiological Findings

Ranking in the top 20 diseases that affect children worldwide (ranking achieved by reporting life years adjusted according to disability), bronchial asthma currently affects approximately 300 million children and adults, with a pediatric mortality rate of 0.7 per 100,000 people and an average annual prevalence of 9.5% among children, which seems to be influenced rather by socio-economic and environmental factors (for example, the quality of the living environment, exposure to active or passive smoking, animal waste from large farms nearby, as well as the existence of barriers regarding the accessibility of obtaining appropriate medical services) rather than geographical location. The tipping of the scales towards the development of this pathology is therefore dictated by a summation between the influence of exogenous factors, the genetic predisposition of the individual, and the impact of the endogenous microbiome, especially in the first part of life. The microbial composition of healthy lungs is characterized by a predominance of bacteria belonging to the species *Bacteroidetes*, *Actinobacteria*, and *Firmicutes*, while viral respiratory infections are associated with *Haemophilus* and *Moraxella* in young children and asthmatic adults, a dysbiosis that maintains inflammatory activity and contributes to bronchoconstriction and hyperreactivity bronchus [5,6,7,8,9]. 

The role played by dysbiosis in the pathogenesis of asthma is thus predictable. Both antibiotics and anti-ulcer drugs, as well as other drugs, negatively impact the intestinal and pulmonary microbiota, the resulting effects deregulating the bidirectional gut–lung axis, with the consequent initiation of hypersensitivity and hyperreactivity to various allergens respiratory and alimentary [5]. The characteristics of the intestinal microbiome seem to be subdivided depending on the geographical area in which the studied population is located. Thus, there is a difference between the microbiome in the western region compared to that of the non-Western population, the difference which is shown to be between 15% and 30% negative ratio of species found among the former. The hypotheses that have been brought into discussion regarding this aspect focus both on microbiome change, the consequences of technological and cultural evolution in the context of industrialization, and the habits’ characteristics of different degrees of subsistence found in non-Western populations (such as hunting and agriculture) [6].

The exogenous microbiome is defined by an omnipresent exposure of the subject, throughout life, to non-self antigens, considering, however, the greater impact it exerts on the individual, especially at the pediatric age. It is well known the existence of the critical window (from the moment of birth until the age of 1 year), during which the interactions between the novice immune system and external microorganisms can lead both to the training of immunity with the induction of tolerance towards certain antigens, as well as to a protective effect, with the decrease in the risk of developing atopy. The paradigm is reversed with aging when the environment becomes more and more a source of antigenic stimulation and infection, and the same microbial exposure that, once protected against the development of diseases, can currently exacerbate their severity [10].

Considering the strong involvement of the microbiome in the normal development of the body, but also the demonstration of the increased incidence of bronchial asthma in early childhood (probably attributed to environmental factors), current research is guided in the sense of understanding the role played by breastfeeding in health and modulating the ratio between beneficial and harmful bacteria found in infants, the similarity between the specific bacteria found in breast milk and those in infants’ feces is already known, an aspect that reveals the impact of natural nutrition on the colonization of the neonatal intestine. Among these bacteria, there are *Bifidobacterium*, *Bacteroides*, *Parabacteroides*, *Blautia*, *Clostridium*, *Collinsella*, and *Veillonella*. The recent discovery of a microbiome specific to breast milk requires further studies. Until now, only the influence of the maternal metabolic state and antibiotics on it is known. Its origin and the enteromammary transport route are still under research [11,12].

## 4. Clinical and Pathogenic Aspects

Asthma, this extremely complex chronic inflammatory condition, is triggered by an immune response materialized in the form of intermittent, reversible obstruction of the lower airways in response to the narrowing of the diameter and the constriction of the smooth muscles following the action of an environmental stimulus, which can overlap or not over a viral upper respiratory infection. From a clinical point of view, most patients with asthma begin to show symptoms before the age of 3, starting with recurrent episodes of wheezing and/or coughing, which associate with respiratory difficulties, triggered by a viral infection of the upper respiratory tract, exposure to allergens, physical activity or weather changes, complaints that yield to bronchodilator drugs. Thus, the diagnosis in children remains a challenge, being mostly focused on major clinical criteria (doctor-diagnosed parental asthma or eczema) or minor (doctor-diagnosed allergic rhinitis, wheezing without viral upper respiratory tract infections, or peripheral eosinophilia ≥4%) until the age of 5 years, from which time the patient is able to perform a pulmonary function test in order to demonstrate bronchospasm. Worthy of emphasis is that the impairment encountered for more than 2 days/week or 2 nights/month defines persistent asthma, a category that must be treated in all age groups with inhaled corticosteroids (ICS) as daily control therapy [13,14].

The pathogenesis of asthma is mainly explained from the perspective of genetic predisposition towards hypersensitivity mediated by IgE to various environmental factors (smoke, pollution, pollen, microbes, mites, mold, emotional stress, and environmental changes). It associates with other atopies, such as allergic rhinitis, conjunctivitis, atopic dermatitis, or food allergies. The basis of asthmatic exacerbations are often viral infections (a peak incidence among children in autumn and spring), hypersensitivity, defective anti-viral immunity (the interferon being correlated inversely proportional to the increase in eosinophilia in the respiratory tract, IL-4, and total IgE), bacterial infections (which affect mucociliary clearance), but also risk factors, individual characteristics of the patient (body mass index, pre-existing lung function), aspects that influence the increase in addressability for hospitalization and thus an economic burden on global health systems [14,15]. 

At the genomic level, the multiple genetic markers and loci associated with susceptibility or protection regarding the development of asthma have been identified as follows:

- An orosomucoid-like 3 (ORMDL3)/gasdermin B (GSDMB) gene locus was associated with childhood-onset asthma as well as higher total serum IgE levels;
- Common single nucleotide polymorphisms (SNPs) of interleukin (IL)-33 and receptor or IL-1RL1 have been associated with atopic asthma;
- The thymic stromal lymphopoietin (TSLP) gene has been identified as protective against the risk of TH2 asthma [16,17,18].

Modern approaches have changed the orientation regarding the main phenotypes of asthma, previously described as non-atopic (intrinsic) and atopic (extrinsic) asthma. Currently, the scientific consensus divides it into two major groups, respect T2-high and non-T2 (T2-low), the differences between the two being presented in Table 1. The literature records the variability of bacterial diversity depending on the asthmatic phenotype. Thus, *Proteobacteria* expansion appears to be inversely correlated with eosinophil levels but is significantly associated with increased neutrophil levels, while non-typeable *Haemophilus influenzae* is detectable during stable disease periods and is associated with low T2 inflammation, increased neutrophils, and resistance to steroids [19,20,21].

In addition to all the above, in growth, the development of the central nervous system, but also in the pathogenesis of asthma and atopic diseases, a central role is occupied by the disturbance of the microbiome–host balance, one of the main determinants of the alteration of immune tolerance and the escalation of asthma severity. It is influenced by a variety of variables, among which the type of birth, breastfeeding, a childhood spent in the countryside, and/or in contact with animals, notions that have been framed across time under the umbrella of the “hygiene theory”, considered a cornerstone foundation regarding the complete elucidation of the physiopathogenesis of atopies. Thus, the importance dictated by the analysis of the composition of the intestinal and respiratory microenvironment, but also of the surrounding environment, in the early prediction, the differentiation of asthma types and the prophylaxis/management of exacerbation crises is emphasized, as its involvement in response to glucocorticoids and certain biological agents is proven [25,26,27,28]. The criteria that must be taken into account regarding the research on the development of the microbiota and the interference between it and the patient’s health are described in Table 2.

### 4.1. Involvement of the Exogenous Environment

Starting from the words of the famous Jean Jacques Rousseau, “study nature and follow the path she shows you”, current research focuses on the influences brought by environmental factors in the development of the most popular pathologies. Consequently, the “hygiene hypothesis” was advanced, starting from the observation of the escalation of atopic diseases in the Western world along with the reduction of microbial exposure during early life by improving sanitation and increasing immunization rates. In contrast, the “biodiversity hypothesis” suggests that reduced exposure during childhood to the rich environmental microbiome associated with natural green spaces prevents appropriate development of the host’s microbial community, thus leading to immune dysregulation through imprecisely defined mechanisms. At the same time, the involvement of endotoxin (the component of the gram-negative bacterial wall) in the modulation of sensitization to allergens was brought into consideration, with a clearly more significant effect in the case of exposure in early life, but also of living in large families, in the company of animals or in farms, while the negative effects of pollution, both in newly identified cases and among those already recorded, both in adults and in the pediatric population, are not to be neglected [29,30,31]. 

In this sense, the “epithelial barrier” of the skin, of the genitourinary, respiratory, or intestinal tract, seems to be influenced by the environment–host interactions, with proven effects of its disruption (by pollution, microplastics, nanoparticles, tobacco smoke, household detergents, and cleaning products, as well as climate change or exposure to environments intensely populated by various fungal species), but also of natural phenomena such as global warming, floods, rising CO_2_ levels, storms (including sandstorms), and wildfires escalation of asthma symptoms [32,33,34]. A cross-sectional study, carried out in two phases (March 2012 and November 2019), which targeted preschool children from the city of Taiyuan, concluded that the internal microbiome is linked to multiple chronic diseases, including atopic diseases, five bacterial genera, and one fungal (*Aspergillus*) being incriminated in association with symptoms of asthma, rhinitis, and eczema in preschool children [35]. 

Lee et al. brought into discussion, with the help of a randomized crossover study focused on the footprint dictated by the use of air purifiers among indoor air pollutants (in particular of suspended particles with an aerodynamic diameter less than or equal to 2.5 μm) and in the control asthma in children, their positive impact on improving medical management by reducing the frequency of used therapeutic products [36]. 

### 4.2. The Infant Microbiome

In addition to environmental factors, individual factors have also proven to be particularly important in the biological course of each person. The beginning of life is a crucial aspect in defining further progress, and in this sense, there is recent evidence that attests that not only the genetic component can tilt the balance but also the microbiome formed by exposure to various stimuli of maternal origin in utero (through the placenta), in the course of birth and breastfeeding. Thus, they came to the conclusion that low biodiversity of the microbial flora during childhood can be associated with subsequent atopic risks, delimiting favorable conditions for the development of an adequate microbiome such as natural birth at the expense of cesarean birth (newborns delivered vaginally being carriers of beneficial bacteria for the regulation of the immune system, while those born by cesarean section show colonization with bacteria specific to the skin (*Streptococcus*, *Staphylococcus*), the preference for breastfeeding in favor of the use of milk formulas, but also the absence of exposure to antibiotic therapy in utero, in the neonatal period, or in the first year of life [37,38,39]. The purpose of the integral development of this bioenvironment resides in its involvement in the activity of regulatory T cells, cells responsible for maintaining in optimal parameters the ratio between T-helper 2 and T-helper 1 lymphocytes, a dynamic fraction, whose impairment induces an increase in the risk of atopic disorders, among which we recall asthma [38].

The interactions between the pregnant woman and the environment modify both her microbiome, as well as the child’s predisposition to develop various diseases, or on the contrary, to improve its immune defense through mechanisms such as the maternal–fetal immune alignment, exposure to metabolites or other bacterial components, bacterial colonization in utero (via the placenta, amniotic fluid, or meconium), but also the vertical transmission of the microbiota at the time of birth [40]. Nutrition during the nine months of pregnancy represents a pillar in the harmonious development of the fetus (intrauterine and extrauterine) but has a weaker impact than breastfeeding. Thus, emphasizing the most important protective factors as being represented by an association between a Mediterranean diet, the consumption of meat, fatty fish, and supplements based on vitamin D, E, copper, zinc, and manganese, while the factors incriminated as having a high risk were pasta and animal products rich in arachidonic acid (e.g., beef/chicken, eggs, butter, organs) [41,42]. 

The diversity of the maternal diet during pregnancy, therefore, represents a key point in the development of the fetus. Dosage of food is vital to avoid both nutritional deficits and over-eating (a risk factor for spontaneous abortion, gestational diabetes, preeclampsia, or wheezing and asthma). The mechanism incriminated in the maternal obesity–asthma relationship is presented by the pro-inflammatory effects exerted by adipokines released from adipocytes but also by possible direct epigenetic effects of obesity [43,44]. Although the studies that correlate its composition with the infant microbiome are poorly represented and are in the initial stages in the human species, Mirpuri J. et al. emphasize the importance of low-fat consumption, contrasting with high-fiber consumption [45]. The Western diet, based on simple carbohydrates, fats, and animal protein, is incriminated in the potentiation of maternal intestinal dysbiosis, described by the increase of *Clostridium innocuum*, *Eubacterium dolichum*, *Catenibacterium mitsuokai*, and *Enterococcus* in contrast to the decrease of *Bifidobacteria* and *Bacteroidetes* [46]. 

It is estimated that the caloric requirement is between 70–500 kcal/day, in addition to the individual one, depending on the trimester of pregnancy. Important micronutrients during pregnancy are represented by iron, calcium, iodine, vitamins (D, C, A), folic acid, and phytochemicals (flavonoids and carotenoids). Iron deficiency predisposes to fetal growth retardation, preterm labor, low birth weight, and postpartum hemorrhage. Additionally, the lack of vitamin D is associated with the risk of preeclampsia, prematurity, and low birth weight, while deficient folic acid has been implicated in neural tube defects [43]. Given the location of the receptors for vitamin D, current studies attest to the positive effect of supplementation in the development of immunity in offspring, fetal lung function, and the reduction of the wheezing breath incident. Similar effects were observed in the case of supplementation with vitamin E, zinc, and omega-3 fatty acids [44,47]. However, the optimal dose of vitamin D administered for the purpose of regulating the immune system has not yet been established. This may be variable compared with the dose required for osteogenesis processes, over and under dosage being the individual inflammatory predisposition and IgE levels [44]. 

The supplementation of iron deficiency in children remains contradictory, this being beneficial in the case of those at risk of anemia but requiring caution due to the risk of favoring the multiplication of harmful bacteria (*Proteobacteria*, *Firmicutes*) or diarrhea (with the growth of *E. coli* to the detriment of the beneficial bacteria among which *Bifidobacteria* or *Lactobacilli*) [48]. On the same note, sodium and magnesium levels in the diet have been a research concern, unfortunately, without concrete results. It started from the hypothesis of their action on the bronchial smooth muscles, the intake of sodium increasing the risk of asthma exacerbation in parallel with that of magnesium, which seems to lead to a decrease in lung function in children, the latter being intravenously administered to control the asthmatic crisis [49]. 

The maternal lifestyle is strongly correlated with the subsequent evolution of the fetus. In the case of our study, we consider it relevant to emphasize the impact of smoking on the increased incidence of lung diseases (airway hyper-reaction, wheezing, asthma, pulmonary ventilation imprinting, and bronchitis) [43]. Other practices such as alcohol consumption, illegal substances, and poor hygiene have been blamed for disrupting the microbial balance at the vaginal, oral or maternal intestinal level, but also at the fetal level [46,50]. In contrast, maternal living in environments specific to agrarian areas decreases the risk of allergies (in particular, asthma) in the offspring [47]. 

The correlation between maternal nutrition and asthma in infants is dictated by the principle of the maternal–fetal immunity alignment, a process achieved by the trans-placental transfer of maternal IgG after the 13th week of gestation. Likewise, microbial metabolites of maternal origin (acetate, propionate, butyrate) exert, transplacentally, an anti-inflammatory (tolerogenic) effect, modulating the capacity for epigenetic programming and energy reserves and thereby imprinting the development of fetal immunity. In this sense, the studies carried out on infants with a familiar history of atopy have concluded the prophylactic effect (for eczema, atopic, or food sensitivities) played by probiotics of the genus *Lactobacillus* and *Bifidobacteria* administered before and after birth, although the recommendation for their routine use still remains contradictory [47,51].

The opinions regarding the supplementation with probiotics during pregnancy are numerous and in a continuous process of research and argumentation, reaching the consensus that the administration of them, both among mothers and children, can reduce the risk of allergies, especially in groups with multiple predisposing factors [40,52].

The type of birth dictates the microorganisms with which the newborn comes into contact. The hypothesis was also supported by Galazzo et al. [53] with the help of a longitudinal study that analyzed stool samples from 440 children from early childhood to school age (11 years). For example, natural birth exposes the fetus to the bacteria found along the birth canal (*Lactobacillus*) with which it comes into contact during labor and expulsion, while, in the case of cesarean birth, a more significant input in modulating the microbiome, it is brought by external factors such as medical equipment, the air, but also the infants and healthcare workers with whom they interact and who can be contaminated with multiple pathogenic or non-pathogenic organisms, for example *Clostridium difficile*. Other than the two types of birth, we also mention the cases of fetuses born via cesarean section following the initiation of labor (“warm caesarean section”), a situation in which it seems that the microbiota tends to be similar to the found in physiological births. Prematurity reflects a significant burden and added risks due to prolonged exposure to the environment, existing in neonatal intensive care units populated by species such as multi-drug resistant *Acinetobacter baumannii*. Thus, unlike full-term children, premature children more frequently present pathogenic microorganisms (*Klebsiella pneumonia*, *Streptococcus*, *Escherichia coli*, and *C. difficile*) and facultative anaerobes (*Enterobacteriaceae* and *Enterococcaceae*) in association with a lower level of anaerobes (*Bifidobacterium*, *Bacteroides*, and *Atopobium*) and short-chain fatty acids. The difference in microbial diversity between preterm and full-term infants appears to be due to maintaining a high level of oxygen in the gastrointestinal tract due to medical practices such as the use of continuous positive airway pressure [53,54,55,56,57].

The World Health Organization (WHO) recommendations follow the exclusive breastfeeding of newborns in the first 6 months of life, with the introduction of complementary food later, up to the age of 2 years or more. Feeding the newborn through breastfeeding (source of immunoglobulins, fatty acids, hormones, and cytokines) is an important process from the point of view of psychological exploration connection between mother and child, as well as in promoting the proliferation of beneficial bacteria in the intestine and the delivery of microorganisms originating from the maternal intestine and the oral cavity of the fetus, describing probiotic and prebiotic effects. Once weaning, the child’s microbiota begins to evolve towards the adult form, with the reduction of *Bifidobacterium* species and the increase in the abundance of *Firmicutes* (*Veillonella*, *Staphylococcus*, *Streptococcus*, *Dolosigranulum*, *Oribacterium*, *Alloiococcus*, *Clostridium*, and *Enterococcus*) among asthmatic children [38,39,58]. Variables that influence the microbiological composition of breast milk are, therefore, antibiotic therapy (reduces *Bifidobacterium* and *Lactobacterium*), the body mass index (BMI) of the mother (inversely proportionally correlated with the level of *Bifidobacterium*), geographical affiliation, but also the oral bacterial flora of the infant that acts through a “retrograde transfer” mechanism [59].

The composition of breast milk varies with the stage of lactation, gestational age, maternal nutritional status, and maternal lipid storage [43,60]. Educating and raising awareness of new mothers (especially those at risk of giving up due to lack of motivation) regarding the importance of achieving optimal breastfeeding both from a nutritional point of view (directly influenced by the maternal diet) and in terms of time is essential in pediatric practice. The nutritional status of the newborn depends on this, but also the risk of developing multiple affections in the future. Regarding the pathology treated in the current study, breastfeeding seems to exert a protective effect regarding the development of asthma in early childhood, an effect that is diminished after the age of 6 [49,60]. The functions of the main constituents in breast milk are diverse and strongly correlated with the mother’s diet and the infant’s homeostasis, including the following:

- Oleic and palmitic acid;
- Lactose is the nutrient source for bacteria such as *Bifidobacteria* and *Lactobacillus*;
- Oligosaccharides (glucose, galactose, fucose, N-acetylglucosamine, N-acetylneuraminic acid, or sialic acid) inhibit the growth of pathogenic bacteria such as *Streptococcus pneumoniae*, *Campylobacter jejuni*, and *E. coli*, and prevent rotavirus by acting as a “bait receptor”; a prebiotic role for *Bifidobacteria*;
- B-group vitamins (B1, B2, B6, B12), vitamin A and vitamin D, sodium, potassium, magnesium, and zinc influence bacterial diversity (e.g., zinc limits the proliferation of intestinal bacterial species);
- Bioactive constituents are those such as antimicrobial substances, growth factors, cytokines, chemokines, anti-inflammatory factors, hormones, digestive enzymes, or transporter substances (lactoferrin, folate ligand, cobalamin ligand, IGF ligand, thyroxine ligand, corticosteroid ligand).

Among the antimicrobial substances, we note lactoferrin, which performs its function by binding iron molecules with high affinity, thus making it unavailable to intestinal bacteria. Other functions are preventing the formation of intestinal biofilm, inhibiting the growth of gram-negative organisms, activating macrophages (phagocytizing gram-positive bacteria), and preventing necrotizing enterocolitis [61,62].

The risk of asthma has therefore been shown to be associated with transient microbial dysbiosis at the intestinal level, in the first 100 days of life, according to the Canadian Longitudinal Study of Healthy Infant Development (CHILD). This disturbance is appreciated by the decrease found in four specific bacterial genera (*Faecalibacterium*, *Lachnospira*, *Veillonella*, and *Rothia*), changes that are less obvious until the age of 1 year, in association with the relative increase of microorganisms such as *Clostridium difficile* and *Clostridium neonatale*. The authors also recall the protective effect on airway inflammation exerted by propionate, butyrate, and acetate (by modulating regulatory T lymphocytes and dendritic cells), the latter being identified in lower concentrations among subjects with asthma [38,63]. 

Last but not least, the sex difference and the impact of maternal asthma during pregnancy were studied, aiming to decrease the population of *Lactobacillus* in the intestinal microbiota of infants born to asthmatic mothers (a negative point being the association of obesity as a comorbidity), especially those of Caucasian origin and male newborns, who also present a higher risk of developing wheezing in childhood, unlike female children [64].

### 4.3. The Gastrointestinal Microbiome

The components of the intestinal environment play an important role in regulating the body’s homeostasis (immunological, metabolic, structural, and neurological variations). The effects appear through its capacity to restrict the development of pathogenic and potentially pathogenic structures, both by inhibiting the invasion capacity and by secreting some bactericidal substances. Variability depends on age, genetic baggage, diet, smoking (active or passive), medication consumption, and various intestinal function constants such as pH, peristalsis, and intestinal transit. Therefore, numerous axes are taking shape that connects the intestinal microbiota to the vital organs (brain, heart, lungs, kidneys, liver, skin), with a crucial impact both in the development and optimal functioning of the individual, as well as in the morbidity induced by childhood asthma [65,66,67,68,69]. Disturbance of the aforementioned axes, observed in the dysbiosis of the intestinal microbiota, can represent the key point in the pathogenesis of a multitude of diseases, among which gastrointestinal diseases (celiac disease, irritable bowel syndrome), cardiac, respiratory, neurological, metabolic, or even allergies and autoimmunity, their knowledge and awareness opening new horizons in the management of organic diseases [70,71].

Following an evolutionary path, the theories regarding the species that colonize the intestine are contradictory, exposing both the existence of age-dependent variability aimed at the different ratio of their abundance during the newborn, infant, child, and adolescent periods than the preferential involvement of certain incriminated genera, as well as the child’s microbiome differs from the adult’s in diversity and stability, the progression between the two stages being marked by the decrease in the number of aerobes and facultative anaerobes, with simultaneous increases in anaerobic species [72,73]. Regarding the impact of dysbiosis occurring in the various stages of life in the development of atopies, it has been recorded in the literature that it shows a modest correlation with asthma when it is developed at preschool age, compared with its appearance in early childhood [74].

Regarding the association of microbial variability with asthmatic phenotypes, the presence of heterogeneity in eosinophilic asthma was objectified, together with a negative correlation between *Proteobacteria*, *Firmicutes*, and pulmonary eosinophilia, while in the neutrophilic form, a lower microbial diversity was noted, with an increased prevalence of potentially pathogenic organisms (*Haemophilus*, *M. catarrhalis*) in association with commensal ones (*Streptococcus*) [75]. In addition, short-chain fatty acids, polyunsaturated fatty acids, and bile acids play a strong role in the pathophysiology of bronchial asthma. As representatives of the class, we find, from the side of short-chain fatty acids, acetate, propionate, and butyrate resulting from the fermentation of dietary fibers. Bile acids are cholic acid and chenodeoxycholic acid, while polyunsaturated fatty acids come under their umbrella omega-3 and omega-6 fatty acids (α-linolenic acid, eicosapentanoic acid, docosahexaenoic acid, linoleic acid, and arachidonic acid). All these metabolic components modulate through their level of the body’s allergic response [76,77]. 

Returning to the most important intestine–body axes’ connection with the lungs, it has been intensively studied in the light of various pathologies that seem to be interconnected, modulating each other. Regarding the way of communication between the two organs, this seems to be performed through prostaglandins, dendritic cells, short-chain fatty acids, and T and B regulatory lymphocytes. Subsequently, naïve immune cells activated in the gut are driven via lymph and blood to eicosatetraenoic the lung, where it exerts effector functions. Thus, the generation of regulatory T cells is modulated. Their early production is associated with protection against long-term allergies [73,78,79,80].

### 4.4. The Respiratory Microbiome

The link between the immune activity of the two organs (intestine and lung) can be explained by the current concept of “common mucosal response”. It involves the association between antigenic presentation in a particular mucosal site and the stimulation of immune cell migration to other sites. Colonization of the upper or lower respiratory tract is different between the oral cavity, nose, pharynx, and bronchi. Thus, the microorganisms incriminated in the increase in incidence, severity, and asthmatic exacerbations proved to be *Moraxella catarrhalis*, *Haemophilus influenzae*, *Streptococcus pneumoniae*, *Pneumocystis*, and *Proteobacteria*. In parallel, the intranasal administration of *Lactobacillus rhamnosus* seems to induce protection against respiratory syncytial virus infection, a pathogen implicated in symptomatic exacerbations [19,81,82,83]. Zhang et al. also certified the dysbiosis occurring in the airways of asthmatics with the help of DNA amplification extracted from the bronchoalveolar lavage fluid obtained from 55 children and the subsequent sequencing of the microbiome [84].

The most intensively studied part of the respiratory tract with regard to microbial colonization is represented by the upper respiratory tract, possibly due to the better accessibility in terms of collecting biological samples from the level of the anterior nostril, the middle meatus, and the nasopharynx, in contrast to the lower part where bronchoscopy is applied in the collection of samples. The components of the innate and adaptive immune system designed to protect against the aggression of exogenous pathogens are represented by the mucus layer rich in lipids and glycoproteins, antimicrobial peptides (lysozyme, lactoferrin, defensins), and reactive oxygen species (hydrogen peroxide, nitric oxide), lymphoid tissue, but also a trigeminal chemesthetic system [85,86]. One of the disadvantages of easy accessibility at this level is represented by the multiple interactions with the external environment, which can overcome immune defense barriers, causing changes in biodiversity, as observed by Ahmed et al. regarding the variables encountered in the colonization of subjects living in the rural environment (*Corynebacterium*, *Staphylococcus*, *Alloiococcus*, and *Peptoniphilus*), in contrast to those from the urban environment (*Staphylococcus*, *Sphingomonas*, and *Moraxella*), attributed to the increased exposure to environmental pollutants [87]. 

Severe asthmatic phenotypes are often accompanied by the presence of comorbidities such as obesity, rhinosinusitis, or gastroesophageal reflux disease or are treated with oral corticosteroids, variables that can influence the composition of the pulmonary microbiota [80,88]. During the first year of life, the composition of the nasopharyngeal microbiome is a determining factor in the occurrence and spread of respiratory infections, increasing the risk of developing asthma in the future. In this stage, in addition to the effects of microaspiration, the elimination of pathogens through coughing and mucociliary clearance, the pH of the environment, the pressure of the airflow, and the presence of certain nutrients favorable for some species, McCauley et al. postulate that the bacterial and fungal nasal microbiota presents a seasonal dynamic, with an escalation of pathogens identified during periods of exacerbation (autumn) [89,90].

## 5. Ways of Investigating the Microbiome

The involvement of the microbiome in the emergence and development of bronchial asthma is therefore evident, with a peak of the impact especially found at the pediatric age (the first year of life) when the novice organism begins to adapt to the external environment, the need to implement collection protocols and study of biological samples, both for the purpose of research and to draw new lines in therapeutic practice. If, with regard to the research techniques, things are already well known, these being represented by 16S rRNA gene sequencing, shotgun metagenomic sequencing, RNA sequencing, proteomics and metabolomics study by mass spectrophotometry electrophoresis, chromatography, and others, Table 3 shows the main sites for collecting the biological samples necessary to study the human microbiome (especially intestinal and respiratory), harvesting techniques, but also the main advantages, disadvantages and mentions in relation to them [91,92,93,94,95].

## 6. Therapeutic Lines

Since the treatment of asthma is not the subject of the current article, we will not insist on the intensively developed therapeutic strategies in the specialized literature. With all this, we want to emphasize the importance of individualized administration of the therapy, both in terms of the combinations and the doses used. These will be evaluated depending on the degree of impairment of each asthmatic patient, as well as the associated comorbidities. The main classes of drugs used in this regard are inhaled corticosteroids (ICS), ICS-formoterol, oral or intravenous corticosteroids, anticholinergics, short-acting beta 2-agonists (SABA), long-acting beta 2-agonists (LABA), leukotriene receptor agonists (LTRA), long-acting antimuscarinic agents (LAMA), non-selective phosphodiesterase inhibitors, mast cell stabilizers, and monoclonal antibodies (Omalizumab, Mepolizumab, Benralizumab, Dupilumab, Tezepelumab) [97,98]. In addition to these, current research emphasizes the importance of administering probiotics, prebiotics, and symbiotics, but also fecal microbiota transplantation in the regulation of the microbiota and in the management of bronchial asthma. To facilitate an easier understanding, we define probiotics as live bacteria from food or supplements intended to enrich the microbiota, while prebiotics represents an additional supply of nutrients administered in order to stimulate and develop beneficial bacteria. To these are added the symbiotics, a mixture of live beneficial bacteria and nutrient substrate for the microorganisms of the host. At the same time, fecal microbiota transplantation represents replacing the patient’s dysbiotic bacterial flora with the bacterial flora of a healthy person.

With reference to the subject of the current narrative work, although difficult to study due to the long period required to observe the patients, we found mentioned in the literature implications of the use of probiotics (in various forms, made up of single strains or combinations of lactic acid bacteria and *Bifidobacteria*) among asthmatic patients. However, we have not found well-established recommendations regarding the administration in order to prevent allergic diseases. In this sense, only suggestions are present in the literature regarding their administration in pregnant women, during breastfeeding or childhood in the population categories considered at risk of developing atopy (with significant hereditary antecedents) [99,100]. With regard to the mode of action of probiotics, we know the role played by them in reducing inflammation and intestinal permeability, as well as increasing IgA objectively in the feces. The value of the latter at the age of 6 months positively correlates with the protection against IgE-mediated atopies [101]. Besides this, probiotics regulate the composition of the microbiota and the integrity of the intestinal barrier, the competitive adhesion to receptors, the expression of junction proteins, and favor the production of bactericidal substances [102].

At the level of the respiratory tract, probiotics have proven their effect in restoring the epithelial barrier destroyed by environmental factors or the administration of antibiotics while also interacting with the microbes present in the human body and preventing the growth of pathogens through the production of antimicrobial substances, competitive colonization, immunomodulation, and change of pH in the niche. There are two forms of them used in medical practice, namely the oral form and the local form in the form of an intranasal spray (under observation due to the potential risk of aspiration that can cause inflammation of the lower respiratory tract) [85]. The oral form of probiotics has questionable effects in the treatment of asthma, but improvements have been observed regarding the frequency of asthma attacks, while the addition of prebiotics has been shown to be beneficial in the prevention of asthma, and symbiotics seem to have an effect in reducing wheezing, the use of asthmatic medication, the management of the condition, but not in the decrease of serum IgE in the studied group, the data in the literature is limited in part by the fact that many studies do not differentiate prebiotics from probiotics and symbiotics [103].

Wang HT et al. draw attention to the difficulties encountered in studying the probiotic–asthma relationship in children, aspects that reside in the coexistence of eczema in this age group, with an increased incidence compared with asthma and which can influence the research results [104]. Another important aspect in the nutrition of pregnant women and small children is following a diet rich in fruits and vegetables (the source of phytochemicals that prevent the development of cardiac, metabolic, malignant diseases and neurodegenerative disorders), supplementing possible nutritional deficiencies, as well as using enriched formulas with oligosaccharides, substances that act on galectin-9, increasing interferon-gamma production by T-helper 1 (Th1) cells and promoting regulatory T cells. In dysbiosis precipitated by antibiotic therapy or cesarean delivery, Korpela K. et al. recommend supplementing infants with a combination of probiotics, doubled by breastfeeding, to support the microbiota. For older children (1–13 years old), fiber is administered in a dose of 5–31 g/day [43,47,62,105].

In addition to prebiotics, probiotics, and postbiotics, the current proposals also discuss vaginal inoculation and fecal microbiota transplantation (FMT), which has the advantage of facilitating a more robust and long-lasting community of beneficial, diverse microorganisms delivered with the help of a single dose, with the risk of insemination accidental contact with pathogenic species or the transfer of resistance to antibiotics [106]. We also draw attention to the need to deepen medical studies regarding the therapeutic possibilities in bronchial asthma, FMT representing an option that is, however, limited as an exploration in the case of this pathology. We thus encourage the focus of future efforts toward deepening awareness in the field of FMT therapy for asthma.

## 7. Conclusions

In conclusion, having an extraordinary variability, the human microbiome remains a subject of strong interest in medical studies, being proven to play a vital role in modulating the body’s homeostasis both by creating a predisposition to the development of various ailments when unbalanced and by forming a true line of defense against external elements, i.e., allergens and environmental factors. Therefore, as we presented above, the microenvironment has various medical implications, atopies representing an area of interest in its interactions. We reported the bidirectional interactions between various organs and the microbiome, the imprint played by the external environment in the balanced development of the child, especially in the first year of life, but also the impact displayed by maternal factors such as birth, breastfeeding and the consumption and/or administration of antibiotics in the prenatal period or perinatal. With references to the treatment of asthma, the pharmaceutical aspects are clearly outlined in the literature, but the impact of the administration of prebiotics, probiotics, symbiotics, fecal matter transplantation, and vaginal inoculation remains open to further scrutiny. The subject is vast and difficult to argue in part because of the extensive follow-ups of patients for an extended period. Therefore, there is the risk of losing a vast amount of information, impacting the results of the outcome despite patients being put under close observation. However, we must continue to make efforts to ensure that the results are truthful and reproducible at all times.

## Figures and Tables

**Table 1:** Major groups of asthma and their characteristics (adapted from Kuruvilla et al. and Akar-Ghibril et al.) [22,23,24].

Pathophysiology	T2-high (atopic)	T2-low (non-atopic)
	Damage to the respiratory epithelium barrier with subsequent changes in: IL-25 and IL-33, with an effect on ILC2, which produce up to 10 times more IL-5 and IL-13 after stimulation; Thymic stromal lymphopoietin (TSLP), with an effect on antigen-presenting cells (dendritic cells) that modulate the expression of T and B lymphocytes, a process that then leads to the production of IL-4, IL-5, and IL-13 cytokines; Eosinophils are attracted to the site of inflammation with the help of chemokines, releasing cytotoxic proteins such as major basic protein (MBP), eosinophil peroxidase (EPX), eosinophil cationic protein (ECP), and eosinophil-derived neurotoxin (EDN); Mast cells and basophils express high-affinity IgE receptors, secreting upon activation histamine and lipid mediators—prostaglandin D2 (PGD2) and Cysteinyl leukotrienes (CysLTs); Serum IgE; PGD2 produces vasodilatation and increased vascular permeability, being associated with a weaker control of the disease and a more severe form of it;	NLRP3 inflammasome activation; Increased IL-1β is characterized by neutrophilic (sputum neutrophils > 40–60%) or paucigranulocytic (i.e., normal sputum levels of both eosinophils and neutrophils) inflammation and a lack of response to corticosteroid therapy; Increased levels of IL-17 (A and F) and IL-8.
Phenotypes	Allergic asthma with early onset. Positive allergen skin tests and increased serum IgE; Late-onset eosinophilic asthma represents an adult condition, presenting a corticosteroid-resistant phenotype with an unknown molecular mechanism; Aspirin-exacerbated respiratory disease, late-onset subtype, occurring as a respiratory reaction induced by COX-1 inhibitors;	Associated with obesity; Associated with smoking; With late-onset.
Clinical correlations	Genetic factors. The ORM1-like 3 (ORMDL3) and gasdermin B (GSDMB) genes located on chromosome 17q21 have been implicated; Viral etiologies, especially rhinoviruses; Air pollution. Ozone, diesel exhaust particles, and suspended particles; Neurogenic inflammation involving transient receptor potential vanilloid 1 (TRPV1) and nerve growth factor (NGF);	The clinical correlations reside in the inclusion of the phenotypes specific to the pathology.
Biomarkers	Increased eosinophils in sputum; Blood eosinophilia; Total serum IgE; Allergen sensitivity panel; Fractional excretion of nitric oxide (FeNO); Serum periostin; Dipeptidyl peptidase 4 (DPP-4); Urinal leukotriene E4 (LTE4);	There are no widely approved markers, the proposed variants being the presence of neutrophilia in the blood/sputum, IL-6, but also metalloproteinase 9 (MM9).
Future directions of study	Bronchial tissue transcriptomics; Blood transcriptomics; Sputum transcriptomics; Blood, breath, and urine metabolomics; ! exhaled breath analysis = breathomics.	

**Table 2:** The determining factors of the development and optimal functioning of the microbiota.

The Type of Microbiome	Interfering Factors
Exogenous	Hygiene; Cleaning products; Living environment; Number of family members; Presence/absence of pets; Pollution; Tobacco smoke; Climate change; Natural phenomena;
Infant	Birth; Breastfeeding; Maternal diet; Prenatal and perinatal exposure to antibiotics; Maternal comorbidities; Sex of the fetus;
Gastrointestinal	Age; Genetic inheritance; Diet; Smoking; Drug therapy (antibiotics, proton pump inhibitors); pH, peristalsis, transit time;
Breathing	Pollution; Various treatments (e.g., corticoid therapy); Associated comorbidities (obesity, rhinosinusitis, gastroesophageal reflux disease); Seasonal variations; Defense mechanisms (mucus layer present in the airways, antimicrobial peptides, reactive oxygen species, cough, mucociliary clearance, pH, air flow); Presence of nutrients favorable to certain species.

**Table 3:** Sites used in microbiome research (adapted from Shah et al. and Abdel-Aziz et al.) [81,96].

Site	Sampling Technique	Notes
Upper respiratory tract	Nasal tamponade or washing	− Non-invasive, acceptable, easy to sample frequently; − predominance of *Moraxella*: increases the risk of exacerbations; − The predominance of *Staphylococcus* or *Corynebacterium* decreases the risk of exacerbations and respiratory diseases; − *Moraxella* in vitro was associated with epithelial lesions and increased expression of inflammatory cytokines;
	Saliva, oral tamponade, or mouthwash	− Non-invasive, acceptable, easy to sample frequently; − May show differences related to sex, pH, and dietary intake; − The results may be biased, depending on the amount of water ingested.
	Sputum (spontaneous or induced)	− May represent the microbiota from the lower respiratory tract; − It can be cross-contaminated with bacteria from saliva or the oral cavity; − Infants with *Moraxella* and *Haemophilus* were more likely to develop recurrent wheezing in childhood.
	Nasopharyngeal mucus	− Six months of life: the predominance of *Staphylococcus* increases the risk of wheezing in childhood; − Two years of life: the rhinovirus associated with the presence of *Moraxella* was associated with childhood asthma.
	Hypopharyngeal aspirates	− One month of life: colonization with *S. pneumonia*, *H. influenza*, *M. catarrhalis* increases the risk of developing recurrent wheezing and asthma in childhood; − One month: *Veillonella* and *Prevotella* were associated with the diagnosis of asthma at six years; − Hypopharyngeal microbiome modulates the effect of azithromycin.
Lower respiratory tract	Bronchoalveolar lavage and suction	− Invasive and presents a risk of cross-contamination during aspiration; − Reveals the growth of *Bacteroides*, *Pneumocystis*, and *Proteobacteria* in the case of asthmatic patients, unlike the control groups.
	Brushing and bronchial biopsies	− Represents the microbiota of the lower respiratory tract, including that associated with mucous membranes; − they are invasive and little used.
Intestine	Fecal matter	
	Rectal tampon	− May present cross-contamination with bacteria present on the skin.

## Data Availability

No new data was generated.
